# Elevating brain GLP‐1 and GIP levels as a treatment strategy for neurodegenerative disorders

**DOI:** 10.1002/alz.089262

**Published:** 2025-01-09

**Authors:** Nigel H Greig, Yun Wang, Barry J Hoffer, Kumar Sambamurti, Debomoy K. Lahiri, Michael A Tones, Margaret M Zaleska, Julie A Mattison

**Affiliations:** ^1^ National Institute on Aging, NIH, Baltimore, MD USA; ^2^ National Health Research Institute, Zhunan, Miaoli County Taiwan; ^3^ Case Western Reserve University, Cleveland, OH USA; ^4^ Medical University of South Carolina, Department of Neurosciences, Charleston, SC USA; ^5^ Indiana Alzheimer’s Disease Research Center, Indianapolis, IN USA; ^6^ Indiana University School of Medicine, Department of Psychiatry, Indianapolis, IN USA; ^7^ Cadre Bioscience, St Louis, MO USA; ^8^ Neuro‐D Consulting LLC, Penn Valley, PA USA

## Abstract

**Background:**

Epidemiological studies report an elevated risk of neurodegenerative disorders, particularly Parkinson’s disease (PD), in patients with type 2 diabetes mellitus (T2DM) that is mitigated in those prescribed incretin mimetics or dipeptidyl peptidase 4 inhibitors (DPP‐4Is). Incretin mimetic repurposing appears promising in human PD and Alzheimer’s disease (AD) clinical trials. DPP‐4Is are yet to be evaluated in PD or AD human studies.

**Methods:**

Incretin mimetics have been evaluated in multiple cellular/animal PD models, including in 6‐hdroxydopamine (6‐OHDA) rats, and have demonstrated efficacy. The clinically approved DPP‐4Is, sitagliptin (*Januvia*) and PF‐00734200 (gosogliptin/*Saterex*) were hence evaluated in the classic 6‐OHDA unilateral medial forebrain bundle rat PD model to evaluate their repurposing potential when administered as human equivalent oral doses. Equivalent doses then were administered to naive nonhuman primates (NHPs) to evaluate whether biomarkers of efficacy in rat could be reproduced in NHPs as a further translational step towards human studies. Pharmacokinetics, DPP‐4 inhibition, GLP‐1/GIP and dopamine levels, together with dopaminergic and neuroinflammatory markers, and GLP‐1/GIP receptor levels were quantified.

**Results:**

Sitagliptin/PF‐00734,200 pre‐ or post‐treatment mitigated 6‐OHDA‐induced dopaminergic neurodegeneration, dopamine level loss and neuroinflammation, and augmented neurogenesis in lesioned substantia nigra pars compacta and in striatum, and reduced classical methamphetamine‐induced rotation in rats. This efficacy associated with 70‐80% plasma and 20‐30% brain DPP‐4 inhibition, and with elevated plasma and brain GLP‐1/GIP levels. Alike plasma/CSF DPP‐4 inhibition and elevated GLP‐1/GIP levels were determined in NHPs administered rat equivalent (human translational) sitagliptin doses. In relation to the drug targets, brain GLP‐1/GIP receptor protein levels were age‐dependently maintained in rodents, preserved in rats challenged with 6‐OHDA, and in humans with PD. Combined GLP‐1+GIP receptor activation in neuronal cultures resulted in neurotrophic/neuroprotective actions superior to single agonists alone – particularly when combined with a DPP‐4I

**Conclusions:**

These studies support further evaluation of repurposing clinically approved drugs that elevate plasma/brain/CSF GLP‐1/GIP as a treatment strategy for neurodegenerative disorders. Incretin mimetics are already in clinical evaluation in PD and AD. Similarly, the repurposing of gliptins warrants evaluation both alone and in combination with an effective incretin mimetic.

